# The effect of serum uric acid concentration on the severity of chronic congestive heart failure

**DOI:** 10.25122/jml-2022-0068

**Published:** 2022-12

**Authors:** Ali Hasan Ismaeel Alshamari, Rossal Kamil Kadhim, Sadiq Jebar Ali AL-Mohana

**Affiliations:** 1 Department of Medicine, College of Medicine, Kufa University, Kufa, Iraq; 2 Department of Medicine, College of Medicine, University of Babylon, Babylon, Iraq

**Keywords:** serum uric acid, uric acid concentration, chronic congestive heart failure

## Abstract

This study aimed to investigate the adverse effects of serum uric acid concentration on the severity of chronic congestive heart failure. One hundred patients with chronic congestive heart failure (several risk factors include hypertension, smoking, and diabetes mellitus) were enrolled in this study (51 females and 49 males). Participants were admitted to Alsader Medical City in Al-Najaf from August 2018 to March 2019. Detailed medical history and complete clinical examination were performed for all patients. The patients' ages ranged from 13–90 years, with a mean of 62.1±15.8 years, and the median was 65 years. No statistically significant age difference was observed between males and females (P-value=0.687). Increased serum uric acid had a directly negative effect on the severity of chronic congestive heart failure and hypertension. High serum uric acid concentration was associated with increased severity of chronic congestive heart failure class NYHA III and NYHA IV and a decrement in ejection fraction of the left ventricle.

## INTRODUCTION

There is a controversy regarding the relationship between chronic congestive heart failure and increased serum uric acid. Studies have assessed the possibility of high serum uric acid concentration as a risk factor for heart failure, hypertension, and acute myocardial infarction directly or indirectly [1]. Several mechanisms, including systemic inflammatory response, renin-angiotensin-aldosterone system (RAAS) activity, endothelin-1 expression of cardiac fibroblasts, the platelet adhesion impairment, and decline of glomerular filtration rate, may lead to the occurrence of hypertrophy left ventricle. Hyperuricemia may affect the prognosis of patients with congestive heart failure (CHF). Identifying the specific role of elevated serum uric acid has been challenging because of its association with established cardiovascular risk factors such as hypertension, diabetes mellitus, hyperlipidemia, and obesity [2–5]. Events in congestive heart failure include the hemodynamic consequences, the neuroendocrine, immune, and inflammatory activation impacting several other systems and tissues with physiological and biochemical derangements [6–9]. Hyperuricemia has been documented as an independent prognostic factor in congestive heart failure [10]. This is supported by the beneficial effect of therapy with xanthine oxidase inhibitors in congestive heart failure [11].

## MATERIAL AND METHODS

This study enrolled 100 patients with chronic congestive heart failure admitted to Alsader Medical City in Al-Najaf from August 2018 to March 2019 (51 females and 49 males). Patients were receiving treatment for chronic heart failure, ischemic heart disease, hypertension, and diabetes mellitus, including angiotensin-converting enzyme inhibitors (ACE), angiotensin receptor blockers (ARBs), beta-blockers (B-blocker), vasodilators, diuretics, metformin, sulfonylureas, and insulin. Medical history and clinical examinations were performed for all patients. Serum uric acid was sent for laboratory measurement by spectrophotometry on the second day of admission. Hyperuricemia was regarded as high when the serum level of uric acid exceeded 0.42 mmol/L (7 mg/dl) in males and 0.36 mmol/L (6mg/dl) in females [12]. In addition, fasting blood sugar and blood pressure were also measured. Echo study by Vivid E9 was used to assess left ventricular function and ejection fraction using the Simpson method by Echo Committee. Heart failure was classified according to New York Health Association (NYHA) classification [13]:

- Class 1: ordinary physical activity does not cause undue fatigue, palpitation, dyspnea, and/or angina.
- Class 2: ordinary physical activity does cause undue fatigue, palpitation, dyspnea, and/or angina.
- Class 3: less than ordinary physical activity causes undue fatigue, palpitation, dyspnea, and/or angina.
- Class 4: fatigue, palpitation, dyspnea, and/or angina occur at rest [13].

*Inclusion criteria:* patients with congestive heart failure with risk factors like smoking, hypertension, and diabetes

*Exclusion criteria:* chronic kidney disease, psoriasis, use of thiazide diuretic and other diuretics, Ciclosporin, pyrazinamide, obesity, hematological malignancies and chemotherapy, a diet like red meat and seafood, glycogen storage disease, lead toxicity, lactic acidosis, and alcohol consumption.

### Statistical analysis

We performed the statistical analysis using SPSS^®^ Software version 23.0. Data were interpreted according to the appropriate statistical method and Pearson's product-moment correlation coefficient. A p-value of <0.05 was considered statistically significant.

## RESULTS

We evaluated the serum uric acid level among 100 patients with congestive heart failure. There were 49% males and 51% females, with ages ranging from 13–90 years, with a mean of 62.1±15.8 years and a median of 65 years. There was no significant difference in age between males and females (P-value=0.687) (Table 1). 25 patients were non-smokers, and 75 were smokers and ex-smokers. In addition, 61 patients had hypertension, 49 had diabetes mellitus, and 79 had ischemic heart disease. A significant relationship was observed between uric acid status and hypertensive patients (P-value=0.033), while diabetes mellitus and ischemic heart disease had no significant value. There was a strong statistically significant relationship between uric acid status and NYHA classification (chi-square=21.65, d.f.=3, P-value<0.001). Serum uric acid was higher in patients with advanced congestive heart failure (64.9%) in NYHA class III and NYHA IV, while 35.1% of congestive heart failure were in NYHA class I and II. We used Pearson's product-moment correlation to investigate the relationship between ejection fraction (%) and uric acid level (mg/dL). There was a significant negative correlation between the two variables (correlation coefficient (R=-0.21, P-value=0.039).

**Table 1:** Age of participants.

Gender	Age (years)			P-value
	Mean±SD	Median	Range	
**Male (49)**	62.8±19.1	64	35–85	0.687
**Female (51)**	61.5±11.7	66	13–90	
**Total (100)**	62.1±15.8	65	13–90	

Table 2 compares the uric acid status with the age, gender, and risk factors of the study participants. A significant relationship was observed between uric acid status and hypertensive patients (P-value=0.033), while diabetes mellitus and ischemic heart disease had no significant value.

**Table 2:** Patient's characteristics and uric acid status.

Characteristics		Uric acid status		P-value
		Normal (n=43)	Hyperuricemia (n=57)	
**Age**		62.4±15.8	61.9±16.0	0.877
**Gender**	Male	20 (46.5%)	29 (50.9%)	0.665
	Female	23 (53.5%)	28 (49.1%)	
**Smoking status**	Non-smoker	13 (30.2%)	12 (21.1%)	0.573
	Smoker	7 (16.3%)	10 (17.5%)	
	Ex-smoker	23 (53.5%)	35 (61.4%)	
**Hypertension**		20 (46.5%)	41 (71.9%)	0.033
**Diabetes mellitus**		22 (51.2%)	27 (47.4%)	0.707
**Ischemic heart disease**		34 (79.1%)	45 (78.9%)	0.988

The mean of serum uric acid was 7.14±1.83 mg/dL (3.30 mg/dL to 11.50 mm).

**Figure 1:**
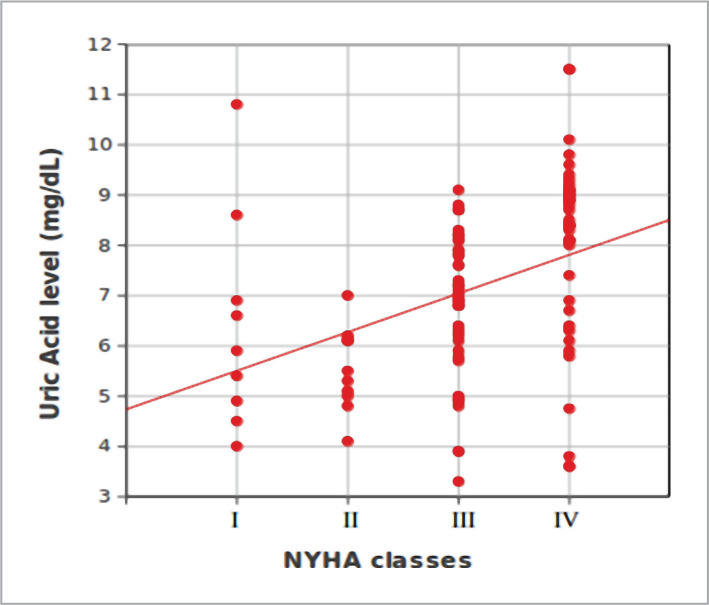
Relationship between uric acid status and NYHA classes.

The relationship between uric acid status and NYHA classes among the study participants is illustrated in Figure 1. There was a strong statistically significant relationship between uric acid status and NYHA classification (chi-square=21.65, d.f.=3, P-value=0.039).

We used Pearson's product-moment correlation coefficient to assess the relationship between ejection fraction (%) and uric acid level (mg/dL). There was a significant negative correlation between the two variables (correlation coefficient (R)=-0.21, P-value=0.039).

## DISCUSSION

This study found that serum uric acid concentration was higher in patients with congestive heart failure (64.9%) and they were in NYHA III and NYHA IV classes. Moreover, 35.1% of hyperuricemia patients with CHF were in NYHA classes I and II. This is similar to Ahmed Khan et al., who identified 58.2% of patients in III and IV NYHA classes, and another study [14] in which patients with III and IV grades of NYHA had an important elevation in serum uric acid. Our study showed a significant negative (adverse) correlation between ejection fraction (%) and uric acid level (mg/dL). This corresponds to Kumbhani DJ [15], who identified a significant negative correlation between serum uric acid concentration and ejection fraction.

**Figure 2:**
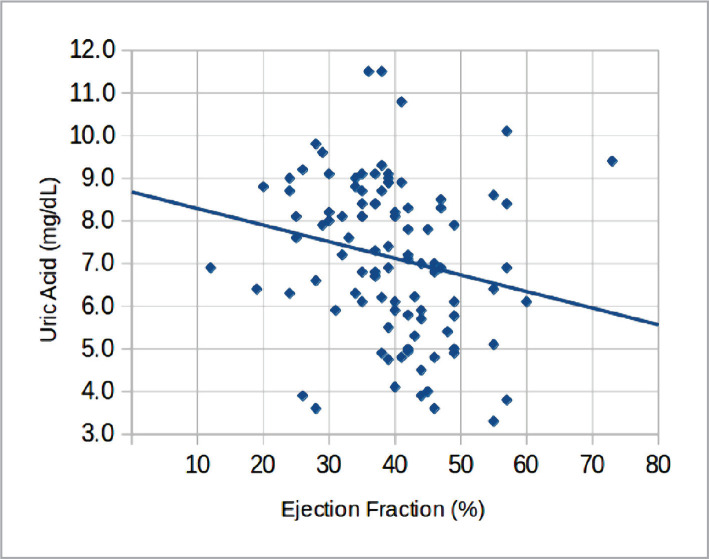
Scatter plot diagram showing the correlation between ejection fraction (%) and uric acid level (mg/dL).

Furthermore, our study revealed a significant relationship between elevated serum uric acid and hypertension (Figure 2). 71.9% of hyperuricemia patients had hypertension compared to 46.5% of normouricemic patients. This finding agrees with Csanz et al. [16], who identified a higher risk of hypertension in the group with higher uric levels and is consistent with other studies [17–19]. This study showed no significant relationship between ischemic heart disease and increased uric acid levels, supported by other studies [20, 21] that argued against this association. Conversely, other studies [22–26] reported an increased risk of ischemic heart disease in patients with hyperuricemia. In this study, the difference in uric acid levels between hyperuricemia and normouricemia patients was not significant, similar to other studies [27–31]. The main limitation of our study was the small sample size.

## CONCLUSION

High serum uric acid concentration was associated with increased severity of congestive heart failure class NYHA III and NYHA IV and a decrement in the ejection fraction of the left ventricle.
